# Functional and Metabolomic Analyses of Chamomile Jelly Derived from Gelatin Capsule Waste with Inulin and Polydextrose as Prebiotic Sugar Substitutes

**DOI:** 10.3390/antiox14111380

**Published:** 2025-11-19

**Authors:** Sasina Sanprasert, Anurak Uchuwittayakul, Pudthaya Kumnerdsiri, Lalitphan Kitsanayanyong, Anusorn Seubsai, Jaksuma Pongsetkul, Kantiya Petsong, Supatra Karnjanapratum, Chalalai Jaisan, Samart Sai-ut, Saroat Rawdkuen, Passakorn Kingwascharapong

**Affiliations:** 1 Department of Fishery Products, Faculty of Fisheries, Kasetsart University, Bangkok 10900, Thailand; sasina.sanp@ku.th (S.S.); pudthaya.k@ku.th (P.K.); ffislhk@ku.ac.th (L.K.); 2 Department of Aquaculture, Faculty of Fisheries, Kasetsart University, Bangkok 10900, Thailand; ffisarb@ku.ac.th; 3 Department of Chemical Engineering, Faculty of Engineering, Kasetsart University, Bangkok 10900, Thailand; fengasn@ku.ac.th; 4 School of Animal Technology and Innovation, Institute of Agricultural Technology, Suranaree University of Technology, Nakhon Ratchasima 30000, Thailand; jaksuma@sut.ac.th; 5 Department of Food Technology, Faculty of Technology, Khon Kaen University, Khon Kaen 40002, Thailand; kantpe@kku.ac.th; 6 Faculty of Agro-Industry, Chiang Mai University, Chiang Mai 50100, Thailand; supatra.ka@cmu.ac.th; 7 Faculty of Agro-Industry, Chiang Mai University, Samut Sakhon 74000, Thailand; chalalai.jai@cmu.ac.th; 8 Department of Food Science, Faculty of Science, Burapha University, Chonburi 20131, Thailand; samarts@go.buu.ac.th; 9 Unit of Innovative Food Packaging and Biomaterials, School of Agro-Industry, Mae Fah Luang University, Chiang Rai 57100, Thailand; saroat@mfu.ac.th

**Keywords:** gelatin, gelatin capsule waste, metabolomic, jelly, inulin, polydextrose, prebiotic, *Lactobacillus plantarum*

## Abstract

Jelly is a popular confectionery, and research increasingly focuses on nutritionally enhanced formulations. In this study, gelatin capsule waste was valorized as a natural gelling base for chamomile jelly, providing an innovative approach to upcycling food-grade waste into functional products. The effects of replacing sugar with inulin (INU) or polydextrose (PDX) (25–100%) on chemical, physical, and sensory properties were investigated. Sugar replacement decreased carbohydrate content while increasing ash and fat, slightly increased turbidity, and reduced lightness (L*) and yellowness (b*). Gels with inulin and polydextrose exhibited higher gel strength (55.97–81.45 g) and hardness (9.77–10.20 N) than the control, whereas antioxidant activity remained largely unaffected. Among the formulations, 50% inulin (INU-50) received the highest consumer acceptance score (6.88 ± 1.05) and maintained stable quality during 21 days at 4 °C, with decreased free water content and increased gel strength. INU-50 jelly supplied essential nutrients, was cholesterol-free, and promoted *Lactobacillus plantarum* growth, supported by metabolomic analysis. Overall, this study demonstrates the potential of chamomile jelly with inulin substitution as a functional, health-promoting product and highlights a novel, sustainable approach to valorize gelatin capsule waste for modern health-conscious consumers.

## 1. Introduction

Jellies are widely consumed due to their attractive appearance, pleasant texture, and palatable taste, particularly among younger consumers [1]. The global market for this product is valued at approximately USD 3.67 billion in 2023, and is projected to grow at a compound annual growth rate (CAGR) of 4.77% through 2033 [2]. Jelly is a type of sugar-based confectionery in which a hydrocolloid matrix forms a three-dimensional network that entraps sugar syrup, resulting in a soft, gel-like texture with relatively high moisture content [3,4]. Common ingredients include hydrocolloids as gelling agents (e.g., gelatin, pectin), sweeteners (e.g., sucrose, glucose syrup), acids, flavorings, and coloring agents [5]. However, their high sugar contents and the inclusion of food additives have raised public health concerns, with excessive and frequent consumption being linked to obesity, dental caries, and hyperglycemia [6]. Furthermore, the limited nutritional value of these products has increased public criticism and driven manufacturers to reformulate jelly products with reduced sugar and more functional ingredients.

In the formulation of healthier jelly products, conventional sweeteners can be substituted with alternative sweeteners and low-digestible carbohydrate (LDC) polymers, such as inulin and polydextrose, to reduce caloric content, improve nutritional quality, and increase dietary fiber intake [6]. Inulin is a fructan-type polysaccharide composed of 3–60 fructose units linked by β-(2 → 1) glycosidic bonds and usually terminated by a glucose residue [6]. It is one of the most widely recognized prebiotics, primarily extracted from chicory root. Nutritionally, inulin provides only about 25–50% of the energy derived from digestible carbohydrates, with a caloric value of approximately 1.39–1.5 kcal/g (6.3 kJ/g) and a sweetness equivalent to ~10% of sucrose [3,7]. These characteristics confer a low caloric content and low glycemic index, supporting its application as a functional ingredient in health-oriented foods. Polydextrose is an amorphous, randomly linked glucose polymer formed through various glycosidic bonds, including α- and β-(1 → 2), (1 → 3), (1 → 4), and (1 → 6), with a certain degree of branching [6]. Owing to its hygroscopic nature, polydextrose functions as a humectant in food systems, while also providing low sweetness, a reduced glycemic index, and a low caloric value of approximately 1 kcal/g [3,8]. Inulin and polydextrose are widely recognized as key functional food ingredients, classified as indigestible polysaccharides and soluble dietary fibers. They are also officially acknowledged as non-digestible carbohydrates with dietary fiber functionality by the United States Food and Drug Administration (USFDA) [6].

Chamomile (*Matricaria chamomilla* L.) is a traditional medicinal herb widely valued for its calming and sedative properties [9]. Its flowers are the primary source of diverse bioactive compounds, including flavonoids (apigenin, luteolin, quercetin) and terpenoids (chamazulene, bisabolol), which contribute to its sleep-promoting effects. The U.S. Food and Drug Administration has classified chamomile as GRAS (generally recognized as safe), confirming its suitability for food applications [10]. Chamomile tea is one of the most widely consumed herbal infusions, with global daily intake estimated to exceed one million cups, and its popularity continues to grow due to its mild taste, refreshing characteristics, and recognized health benefits [11]. Beyond traditional teas and supplements, these attributes highlight chamomile’s potential for development into innovative functional food products, such as jelly, that align with current consumer preferences for convenient and health-promoting foods.

Gelatin capsule waste, a by-product from the soft gel capsule industry, represents a significant underutilized resource [12]. Despite being unsuitable for direct reuse due to processing-induced alterations in its structure and functionality, this material contains substantial nutritional components, with protein accounting for approximately 50–70% of its composition [13,14,15]. These characteristics highlight its potential for valorization in the development of functional food products. Therefore, this study aimed to develop functional chamomile jellies using GCW as a sustainable gelling agent. Inulin and polydextrose were incorporated at varying levels to investigate their effects as sugar substitutes and prebiotic dietary fibers on the product’s physicochemical, functional, and sensory characteristics. We hypothesized that incorporating inulin and polydextrose as prebiotic sugar substitutes into gelatin capsule waste–based chamomile jelly would improve its physicochemical, sensory, and biofunctional properties, supporting its potential as a sustainable functional food product. This approach not only promotes waste valorization but also supports the development of functional foods aligned with the United Nations’ Sustainable Development Goals (SDGs).

## 2. Materials and Methods

### 2.1. Materials

Carrageenan was purchased from Thai Food and Chemical Co., Ltd. (Samut Prakan, Thailand). Inulin and polydextrose were purchased from Siam Ultimate Laboratories Co., Ltd. (Bangkok, Thailand). Gelatin capsule waste was kindly provided by Service Manufacturing Co., Ltd. (Bangkok, Thailand). The *Lactobacillus plantarum* TISTR 1465 strain was obtained from the Microbiological Resource Center, Thailand Institute of Scientific and Technological Research (TISTR), Pathum Thani, Thailand.

### 2.2. Chamomile Tea Jelly Preparation

The chamomile tea jelly was prepared following a standardized procedure. Briefly, dried chamomile flowers (10 g) were infused in 1000 mL of boiling water for 5 min. Subsequently, the flowers were subsequently removed, and the resulting chamomile tea was collected as the base solution. Thereafter, the tea was mixed with other ingredients according to the formulation presented in Table 1. The mixture was heated to 80–90 °C with continuous stirring until complete dissolution and homogeneity were achieved. The hot mixture was then poured into heat-resistant plastic cups and allowed to set under refrigeration at 4 °C until further use. Treatment groups were designated as follows: INU-25, INU-50, INU-75, and INU-100 represented jelly samples in which sucrose was partially replaced with inulin at levels of 25%, 50%, 75%, and 100% (*w*/*w*), respectively; PDX-25, PDX-50, PDX-75, and PDX-100 represented jelly samples in which sucrose was partially replaced with polydextrose at levels of 25%, 50%, 75%, and 100% (*w*/*w*), respectively.

### 2.3. Phase I: Study of Physicochemical and Functional Properties of Chamomile Jelly Products with Sugar Substituted by Prebiotics

#### 2.3.1. Proximate Analysis

The proximate composition (fat, protein, ash, and moisture contents) was determined following the standard methods of AOAC (2019).

#### 2.3.2. Water Activity and pH

The water activity was measured using a water activity meter (Model 4TE; AQUALAB; Pullman, WA, USA). The pH of each chamomile tea jelly sample was determined by dispersing 1 g of the sample in 100 mL of distilled water, heating the suspension to 60–70 °C, and subsequently cooling it to 25 °C. Then, the pH value was measured using a pH meter (Starter 3100; OHAUS, Parsippany, NJ, USA).

#### 2.3.3. Color Measurement

The color of each chamomile tea jelly sample was analyzed using a colorimeter (UltraScan VIS; HunterLab, Reston, VA, USA). The CIE L*, a*, and b* values were recorded, where L* indicates lightness, a* indicates the red–green coordinate, and b* indicates the yellow-blue coordinate. The total color difference (ΔE*) was recorded, and its calculation followed the method described by Anyiam et al. [16].

#### 2.3.4. Texture Profile Analysis

Texture profile analysis (TPA) of each chamomile tea jelly sample was conducted using a texture analyzer (TA.XT Plus, Stable Micro Systems; Surrey, UK) equipped with a cylindrical aluminum probe (P/50). The analysis was performed in TPA mode with a pre-test speed of 1 mm/s, a test speed of 5 mm/s, and a post-test speed of 5 mm/s, applying 50% strain. The parameters obtained were hardness, adhesiveness, springiness, cohesiveness, gumminess, and chewiness.

#### 2.3.5. Gel Strength Determination

The gel strength of each chamomile tea jelly sample was measured using a texture analyzer (TA.XT Plus, Stable Micro Systems, Surrey, UK) with a cylindrical probe (P/0.5), following the Gelatin Bloom method of the Gelatin Manufacturers Institute of America (GMIA) at a test speed of 1 mm/s and a penetration distance of 4 mm. Gel strength, which indicates the firmness of gelatin, was expressed as the Bloom value (g), with higher values representing stronger gels.

#### 2.3.6. Total Phenolic Content and Antioxidant Activity (ABTS•^+^ and DPPH• Assays)

The total phenolic content (TPC) and antioxidant activity of each chamomile tea jelly sample were determined according to the method of Kumnerdsiri, Sanprasert, Praiboon, Seubsai, Sirisarn, Pongsetkul, Harnkarnsujarit, Rawdkuen, Karnjanapratum, Sai-Ut and Kingwascharapong [12]. The TPC was analyzed using Folin–Ciocalteu reagent (FCR), and the results were expressed as milligrams of gallic acid equivalent per 100 g of jelly (mg GAE/100 g). The antioxidant activity was evaluated using two assays: (i) the ABTS•^+^ radical cation assay, with ABTS and Trolox as reagents; and (ii) the DPPH• radical assay, using a DPPH radical solution. The antioxidant activities were expressed as milligrams of Trolox equivalent per 100 g of jelly (mg TE/100 g).

#### 2.3.7. Sensory Evaluation

Sensory evaluation was conducted using 30 untrained panelists, consisting of students and staff from the Department of Fishery Products, Kasetsart University, Bangkok, Thailand. Each panelists evaluated the appearance, color, odor, flavor, taste, texture, and overall liking of each chamomile tea jelly samples using a nine-point hedonic scale (from 1 = dislike extremely, through 5 = neither like nor dislike, up to 9 = like extremely). Prior to serving, each sample was labeled with a random three-digit code and stored at 4 °C until testing. The panelists were instructed to rinse their mouths with room temperature water between evaluations. Ethical approval for this study was granted by the Research Ethics Committee of Kasetsart University, Bangkok, Thailand (COE No. 67/063), under study code KUREC-HSR67/019.

#### 2.3.8. Nutrition Profile

The nutritional composition of selected chamomile tea jelly sample was analyzed using AOAC (2023) methods: ash (923.03), protein (991.20), fat (2003.05), moisture (925.45), cholesterol (994.10), total sugar (982.14), and sodium and potassium (984.27). The calorie content and total carbohydrates were calculated.

### 2.4. Phase II: Evaluation of Physicochemical and Functional Changes in Chamomile Jelly During Storage for 21 Days

The chamomile jelly samples in which sugar was substituted with prebiotics, and which were selected based on consumer acceptance in Phase I, were stored at 4 °C for 21 days and analyzed for their chemical and physicochemical properties. Control samples without prebiotics were included for comparison. Each jelly sample was packed in a round polypropylene (PP, food-grade) plastic cup (7.6 cm diameter × 4 cm height) with a tightly sealed lid to minimize exposure to oxygen and light during storage. Control samples without prebiotics were included for comparison.

Selected parameters (pH, color, texture profile analysis, gel strength, and sensory evaluation) were evaluated using the methods described in Phase I (Section 2.3). In addition, syneresis, In vitro prebiotic activity and microbial assessment were evaluated as described below:

#### 2.4.1. Syneresis

The syneresis was calculated based on a modification of the method by [17]. Each 30 mL sample was placed in a 50 mL centrifuge tube, and its initial mass (m_1_) was recorded. Samples were refrigerated at 4 °C for 18 h and centrifuged at 5000 rpm for 10 min at 4 °C. After carefully removing surface water, the tube was weighed again (m_2_). Syneresis was calculated using:

$$\%Syneresis=\frac{m_{1}\;-m_{2}}{m_{1}}\times 100$$

#### 2.4.2. Microbial Assessment

Microbiological analysis was conducted following the in-house methods of the Food Quality Assurant service center (FQA LAB) with ISO/IEC 17,025 certification. Yeasts, molds, and pathogenic bacteria including *Escherichia coli* and *Staphylococcus aureus* were analyzed following the FDA Bacteriological Analytical Manual (BAM) guidelines. Sampling was conducted at 7-day intervals over a 21-day period.

### 2.5. Phase III: Evaluation of Biological Properties of Chamomile Jelly Products

Chamomile jelly samples in which sugar was substituted with prebiotics and selected based on consumer acceptance in Phase I were further analyzed to evaluate their potential health-promoting effects. The evaluation focused on in vitro prebiotic activity toward *Lactobacillus plantarum* TISTR 1465 and profiling of key metabolites produced during bacterial growth. The detailed methodologies for these analyses are described in the following subsections, providing insights into the biological functionality of the developed chamomile jelly products.

#### 2.5.1. Analysis of In Vitro Prebiotic Activity

The prebiotic activity of chamomile jelly formulations was evaluated based on the growth of *Lactobacillus plantarum* TISTR 1465. The strain was activated in MRS broth at 37 °C for 18 h and adjusted to ~10^6^ CFU/mL. To assess whether inulin or other jelly components could serve as alternative carbon sources, nine media were prepared as follows: Control (chamomile jellies containing 8.57% sucrose), INU-50 (chamomile jelly containing 4.29% sucrose + 4.29% inulin), GCW (5.15% gelatin capsule waste in water), GCW + Chamomile tea (5.15% GCW in 85.71% chamomile tea), GCW + Carrageenan (5.15% GCW + 0.57% Carrageenan in water), GCW + Sugar (5.15% GCW + 4.29% sucrose in water), GCW + Inulin (5.15% GCW + 4.29% inulin in water), MRS broth (positive control), and PBS (negative control). All media were inoculated with the prepared *L. plantarum* suspension (~10^6^ CFU/mL) at a ratio of 1:1 (*v*/*v*) and incubated at 37 °C in a shaking incubator set at 120 rpm for 18 h. Cultures were serially diluted, spread onto MRS agar, and incubated at 37 °C for 48 h. Bacterial growth was enumerated and expressed as CFU/g. The resulting mean value from triplicate samples collected from each experimental medium was subjected to statistical analysis to compare the growth-promoting effects of jelly components.

#### 2.5.2. Metabolomic Analysis

Based on the results of in vitro prebiotic activity (Section 2.5.1), the culture media from the Control (chamomile jellies containing 8.57% sucrose) and INU-50 (chamomile jelly formulation in which sucrose was fully replaced with 50% inulin) groups were selected for metabolomic profiling. After 18 h of incubation at 37 °C, the samples were immediately chilled on ice and centrifuged at 4 °C, 5000× *g* for 10 min to remove bacterial cells. The resulting supernatants were further clarified by a second centrifugation at 4 °C, 16,000× *g* for 10–15 min and then passed through 0.22 µm syringe filters into pre-chilled microcentrifuge tubes. The filtered supernatants were stored at −40 °C until metabolite analysis.

Metabolite extraction and mass spectrometry detection were conducted by Biomarker Technologies (Beijing, China) using Ultra-High Performance Liquid Chromatography-Mass Spectrometry (UHPLC-MS) consisting of a Waters Acquity I-Class PLUS coupled with a Waters Xevo G2-XS QTof high-resolution mass spectrometer. Metabolites were separated on an ACQUITY UPLC CSH C18, 1.7 µm, 2.1 × 100 mm with CSH VanGuard guard. For both positive and negative ion modes, the mobile phases consisted of a 60% acetonitrile aqueous solution containing 10 mM ammonium acetate and 0.1% formic acid as mobile phase A, and a 90% isopropanol–acetonitrile solution containing 10 mM ammonium acetate and 0.1% formic acid as mobile phase B. The injection volume was 5 μL, and the column temperature was maintained at 55 °C throughout the analysis to ensure chromatographic stability and reproducibility.

Mass spectrometric data were acquired in MSE mode under the control of the MassLynx v4.2 software (Waters, Milford, MA, USA). In each acquisition cycle, data were collected simultaneously at low and high collision energies to obtain comprehensive MS^1^ and MS^2^ information. The low collision energy was set to 2 V, while the high collision energy was ramped from 10 to 40 V. The scanning frequency was 0.2 s per spectrum with a mass-to-charge ratio range of 50–2000 *m/z*. The optimized electrospray ionization (ESI) parameters were: capillary voltage of +2500 V in positive ion mode and −2000 V in negative ion mode; cone voltage of 30 V; ion source temperature of 120 °C; desolvation gas temperature of 550 °C; desolvation gas flow rate of 900 L/h, and cone gas flow rate of 50 L/h. These settings ensured efficient ionization and minimized signal suppression during the acquisition process.

Each batch included pooled quality control (pooled QC) injections every 8–10 samples, process blanks, and solvent blanks, with randomized injection order. Isotope-labeled internal standards were added before extraction. Features were retained only if the coefficient of variation (CV) in pooled QCs was ≤30 percent, detected in at least 80 percent of samples in one group, and above blank-derived limits. The limit of detection (LOD) was defined as a signal-to-noise ratio (S/N) ≥ 3 or mean blank + 3 standard deviations (SD); the limit of quantification (LOQ) as S/N ≥ 10 or mean blank + 10 SD. Mass accuracy, retention stability, and drift were assessed using lock-mass calibration and QC-based locally estimated scatterplot smoothing (LOESS); drifted features were removed.

The raw LC-MS data were processed using the Progenesis QI software (Waters Corporation; Milford, MA, USA) for peak detection, deconvolution, and alignment. Metabolite identification was performed by matching experimental spectra against the METLIN database and Biomarker Technologies’ in-house library. Theoretical fragment identification and mass deviation thresholds were controlled within 100 ppm to ensure high-confidence annotation. The raw peak area data were normalized to the total ion signal of each sample to minimize technical variations before statistical analysis.

Annotated metabolites were further classified and mapped to metabolic pathways using the KEGG, HMDB, and LipidMaps databases. Fold-change (FC) values and Student’s *t*-tests were used to determine significant differences between groups.

### 2.6. Statistical Analysis

A completely randomized design (CRD) was used for this study. All experiments were conducted in triplicate (*n* = 3). Data analysis was performed using analysis of variance (one-way ANOVA) with the SPSS software package (SPSS 23.0 for Windows, SPSS Inc., Chicago, IL, USA). The significance of the means was determined using Duncan’s test with a confidence level of 95% (*p* < 0.05). Sensory data were analyzed using a randomized complete block design (RCBD) and Duncan’s multiple range test, with significance tested at the *p* < 0.05 level.

## 3. Results and Discussion

### 3.1. Phase I: Physicochemical and Functional Properties of Chamomile Jelly Products with Sugar Substituted by Prebiotics

#### 3.1.1. Proximate Analysis

The proximate compositions of the chamomile jelly products substituted with inulin and polydextrose at different concentrations are presented in Table 2. Based on these results, the control sample showed the lowest fat and ash contents but the highest carbohydrate content compared to samples with sugar substituted by inulin and polydextrose. (*p* < 0.05). The presence of inulin and polydextrose resulted in a progressive increase in the fat and ash contents with increasing substitution levels, whereas the carbohydrate content tended to decrease. Generally, the determination of the carbohydrate content in food products is calculated based on differences. Therefore, the substitution of sucrose (a fully digestible carbohydrate) with inulin and polydextrose (classified as dietary fibers or low-digestible carbohydrates) resulted in a reduction in the calculated carbohydrate content. This finding was in line with the report of Zainol et al. [18], who demonstrated that the use of dietary fibers or low-digestible carbohydrate-based sweeteners reduced the available carbohydrate content in sweet corn jam (*Zea mays* var. *saccharata* Bailey). Similarly, Lightowler et al. [19] and Do Carmo et al. [20] reported that inulin from chicory root and polydextrose were categorized as non-digestible carbohydrates (NDCs), which do not provoke a glycemic response (GR).

The increased ash and protein content may be attributed to the presence of trace minerals and protein in commercial inulin and polydextrose, which are introduced during the extraction or synthesis processes [21,22]. Typically, these include macroelements such as magnesium, calcium, sodium, and potassium, as well as microelements such as manganese and iron [23]. Upon combustion, these mineral residues contribute to a higher ash content, while sucrose, being a relatively pure compound, contains only minimal mineral impurities. Generally, in commercial products, chicory root-derived inulin contains 0.05 ± 0.00% ash, 0.33 ± 0.10% fat, and 0.43 ± 0.05% protein [21], whereas typically, commercial polydextrose contains 0.50 ± 0.00% ash and 0.10 ± 0.00% protein [22]. Consistent with these results, Zainol, Cheang, Zuraidah, Yahya and Zin [18] reported that the substitution of 9% inulin in sweet corn jam increased the ash content from 0.55% to 0.58%. Likewise, Rodriguez-Huezo et al. [24] found that the addition of 6–18% polydextrose to semolina pasta significantly increased the ash content.

Regarding the fat content, fat content in jelly samples substituted with inulin and polydextrose may be attributed to the ability of certain dietary fibers to disperse in or entrap lipids within their structure [25]. Thus, residual fat from the gelatin capsule waste may have been retained within the fiber matrix, preventing its degradation or loss during thermal processing, leading to higher fat values compared to the control. This aligned with Ferreira et al. [26], who demonstrated that substituting 15% of sugar with inulin in cereal products significantly increased the fat content from 0.2% to 0.4%, suggesting that inulin may act as a fat-retention agent. Similarly, Yang et al. [27] reported that the substitution of 2.5–10% polydextrose in snack products increased the fat content from 29% to 31–35%, while Han et al. [22] also observed higher fat levels in corn-flavored snacks enriched with inulin and polydextrose compared to the control.

Therefore, the incorporation of inulin and polydextrose in chamomile jelly products directly influenced the proximate composition, particularly increasing the fat and ash contents while reducing the carbohydrate content.

#### 3.1.2. Water Activity (*aw*)

Water activity (*aw*) is a critical factor in food systems, as it directly influences microbial growth, chemical reactions, and ultimately product quality [28]. The water activity of chamomile jelly products in which sucrose was substituted with inulin and polydextrose at different concentrations is presented in (Supplementary Table S1). The results showed that the water activity values of the products ranged from 0.9822 to 0.9868. Notably, the control sample exhibited significantly lower water activity than jelly samples with sugar substituted by inulin and polydextrose (*p* < 0.05). This difference may be attributed to the structural characteristics and the number of hydroxyl (−OH) groups present in sucrose versus polydextrose and inulin. Sucrose, as a disaccharide, possesses fewer −OH groups, which limits its hydrogen-bonding capacity with water molecules. In contrast, polydextrose and inulin are long-chain polysaccharides composed of multiple fructose and glucose units, resulting in a substantially higher number of −OH groups. Such structural features enhance their hydrogen-bonding interactions with water, thereby imparting greater hygroscopicity and higher water-retention capacity compared to sucrose. Consequently, products containing inulin and polydextrose exhibited higher water activity values (*aw*) than those formulated with sucrose [3,29]. Typically, water activity values of soft jelly products range between 0.80 and 0.88 [30]. However, aw in jelly systems can vary depending on cooking temperature and formulation [28]. Siriwattanasilp et al. [31] reported that coconut milk jelly with different levels of sweetener substitution exhibited *aw* values between 0.9746 ± 0.0038 and 0.9916 ± 0.0053. Similarly, Chheng et al. [32] demonstrated that carrageenan jelly incorporated with Sesban flower extract had *aw* values ranging from 0.988 to 0.994. In contrast, Mieszkowska and Marzec [33] reported that short-dough biscuits with sugar substituted by inulin and polydextrose exhibited lower water activity than those formulated with sucrose. These findings suggest that the substitution of sucrose with inulin and polydextrose significantly alters the water activity of jelly products, which may subsequently influence their quality and stability during storage.

#### 3.1.3. pH Value

pH plays a crucial role in the formation and stability of gels. Generally, gels exhibit maximum stability near their isoelectric point, as pH directly affects the electrical charge of molecules and the intermolecular interactions among polymer chains. These factors, in turn, influence the aggregation, alignment, and density of the gel network. Therefore, controlling pH is essential for achieving gels with the desired strength and stability [34]. The pH values of chamomile jelly products in which sucrose was substituted with inulin and polydextrose at different concentrations are presented in Table 3. The results showed that the pH of all samples ranged between 7.0 and 8.0. Notably, the control sample exhibited significantly higher pH values than jelly samples with sugar substituted by inulin and polydextrose (*p* < 0.05).

This observation may be explained by the fact that inulin and polydextrose, as oligo-/polysaccharides, can undergo partial hydrolysis or cleavage of glycosidic bonds under heating and moisture conditions during jelly processing. Such degradation may yield weakly acidic compounds, including fructose or organic acid derivatives, leading to a decrease in pH. In contrast, sucrose, a relatively stable disaccharide, is less prone to acid formation under similar conditions [35]. In line with this, Keskin Kuzey et al. [36] reported that the pH of gel-based confectionery products varies depending on the type of oligosaccharide incorporated. Similarly, Delgado and Bañón [35] found that gels containing inulin had lower pH values compared to starch-containing gels. These findings suggest that substituting sucrose with inulin and polydextrose significantly affects the pH of chamomile jelly products.

#### 3.1.4. Appearance and Color

**Figure 1:**
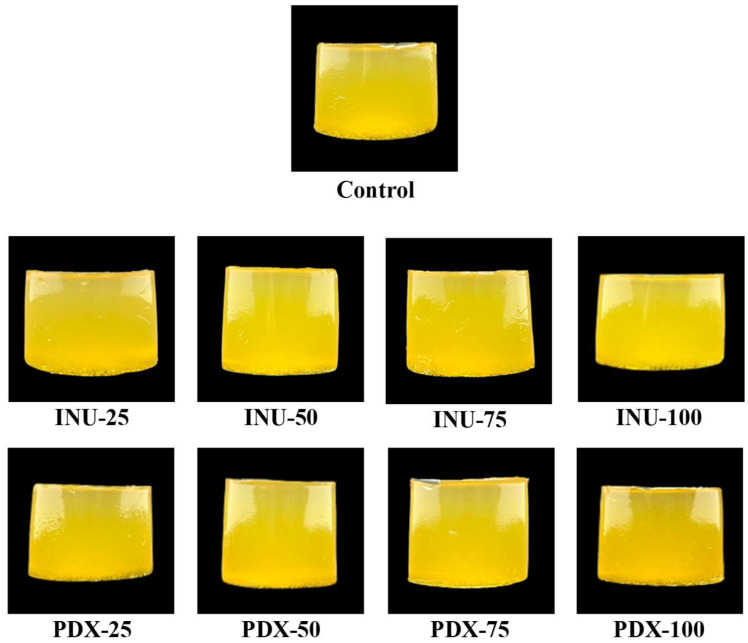
The appearance of chamomile jellies substituted with inulin or polydextrose at different levels. Note: INU-25, INU-50, INU-75, and INU-100 refer to chamomile jellies in which sugar was substituted with inulin at levels of 25%, 50%, 75%, and 100%, respectively, while PDX-25, PDX-50, PDX-75, and PDX-100 refer to chamomile jellies with sugar substituted by polydextrose at the same levels.

The color of jelly products is a crucial factor influencing consumer acceptance, as it directly affects flavor perception [37]. The chamomile jelly product formulated with inulin and polydextrose as sugar substitutes exhibited a yellowish color (Figure 1), which primarily derived from the inherent color of chamomile, the main ingredient in the formulation. During the heating process, the color of the jelly became considerably more intense, which may be attributed to non-enzymatic browning reactions [38]. The color values of chamomile jelly with different concentrations of inulin and polydextrose are presented in Table 3. The lightness (L* value) and the b* value of the control sample were higher than those of the samples containing inulin and polydextrose. This may be explained by the structural properties and the natural color of sucrose, which is characterized by pure white crystalline form and high thermal stability, thus having little effect on product color. In contrast, inulin and polydextrose generally appear as off-white to light brown powders, which may contribute to a lower L* value from the beginning. Moreover, the incorporation of inulin and polydextrose slightly increased the turbidity of the jelly compared with the control. This could be due to the denser gel network structure and altered light scattering, resulting in darker appearance and reduced L* values. On the other hand, no significant differences were observed in the red–green chromaticity (a* value) among the samples, which may be explained by the absence of ingredients in the formulation influencing the a* value.

To better illustrate the overall color variation, the total color difference (ΔE*) was calculated. As shown in Table 3, jellies containing inulin (INU-25 to INU-100) exhibited relatively low ΔE* values (2.12–2.36), indicating that the color differences from the control were minor and likely imperceptible to the naked eye (ΔE* < 3). In contrast, samples containing polydextrose (PDX-25 to PDX-100) displayed significantly higher ΔE* values (4.59–4.87), reflecting clearly noticeable color differences compared with the control. The greater ΔE* values observed in polydextrose-substituted jellies could be attributed to enhanced Maillard reactions or caramelization during heating, as polydextrose is a glucose polymer that may undergo thermal degradation more readily than inulin [39,40,41]. These findings suggest that inulin substitution exerts minimal impact on jelly color, whereas polydextrose substitution leads to more pronounced color darkening.

#### 3.1.5. Texture Properties

The texture of jelly products is a multifactorial attribute determined by raw material characteristics, formulation ratios, and processing conditions. Among these, water content is the most critical factor, as it directly governs hardness, elasticity, and gel stability, thereby shaping overall textural quality [4].

Overall, chamomile jelly substituted with inulin and polydextrose at varying concentrations exhibited higher values of hardness, adhesiveness, springiness, gumminess, and gel strength, while cohesiveness was slightly lower compared to the control samples (Table 4). These improvements can be attributed to multiple mechanisms. First, inulin and polydextrose are long-chain polysaccharides rich in hydroxyl (−OH) groups, which enable strong hydrogen bonding with water molecules and with polymers in the gel matrix, resulting in a denser and more stable gel network. Additionally, the hydroxyl groups of inulin and polydextrose may establish synergistic interactions with the amino and carboxyl groups of gelatin, reinforcing the gel structure and contributing to a firmer and more stable texture compared with sucrose-based formulations. These findings align with [42], who reported that the hardness, adhesiveness, and gumminess of gummy candies increased with higher levels of polydextrose, isomalto-oligosaccharides, and fructo-oligosaccharides.

#### 3.1.6. Total Phenolic Content and Antioxidant Activity

The antioxidant activity of chamomile jelly products with sucrose replacement by inulin and polydextrose at various concentrations is presented in (Supplementary Table S1). The total phenolic content (TPC) and radical scavenging activities (DPPH and ABTS^•+^) were not significantly influenced by the substitution of sugar with inulin or polydextrose. The TPC ranged from 133.69 ± 5.51 to 137.88 ± 1.61 mg GAE/g sample, ABTS^•+^ activity ranged from 2.63 ± 0.08 to 2.75 ± 0.36 µmol TE/g sample, and DPPH activity ranged from 0.11 ± 0.01 to 0.13 ± 0.01 µmol TE/g sample. This finding is consistent with the report of Aamir et al. [43], who observed a comparable total phenolic content (136.92 ± 0.06 mg GAE/L) in a carbonated beverage fortified with chamomile herbal extract. Moreover, Castañeda-Saucedo et al. [44] reported that the total phenolic content (TPC) ranged between 30.22 and 38.27 µg GAE/mL, whereas the ABTS^•+^ and DPPH radical scavenging activities were 0.14–0.20 µmol TE/mL and 0.19–0.75 µmol TE/mL, respectively. This could be attributed to the fact that these sugar replacers are polysaccharides lacking functional groups with intrinsic antioxidant potential, such as phenolic or flavonoid moieties. Consequently, increasing the proportion of inulin or polydextrose in the jelly formulation did not contribute additional antioxidant compounds to the gel system. The antioxidant activity of the products was primarily attributed to chamomile itself, which served as the main determinant of the radical scavenging activity. Therefore, variations in the levels of inulin and polydextrose had no significant influence on the antioxidant capacity of chamomile jelly.

#### 3.1.7. Consumer Acceptance

**Figure 2:**
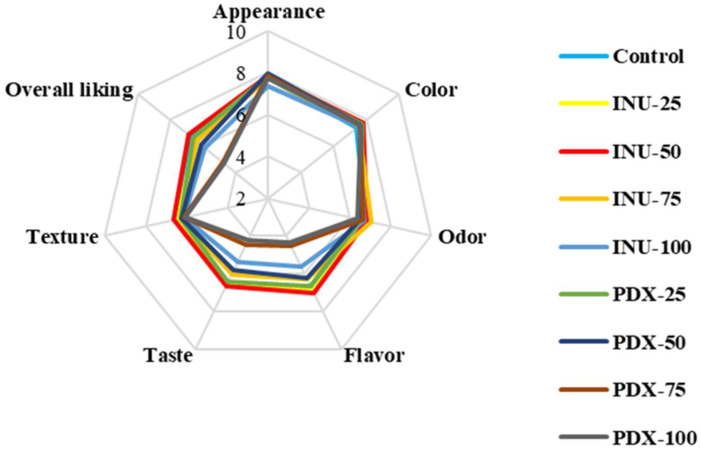
Sensory evaluation of chamomile jellies substituted with inulin or polydextrose at different levels. Note: INU-25, INU-50, INU-75, and INU-100 refer to chamomile jellies in which sugar was substituted with inulin at levels of 25%, 50%, 75%, and 100%, respectively, while PDX-25, PDX-50, PDX-75, and PDX-100 refer to chamomile jellies with sugar substituted by polydextrose at the same levels.

Consumer acceptance scores of chamomile jelly products formulated with varying concentrations of inulin and polydextrose as sucrose replacers are illustrated in Figure 2. Overall, replacing sucrose with inulin or polydextrose tended to reduce consumer acceptance, particularly at higher concentrations of polydextrose, where bitterness became perceptible. The control (100% sucrose) had the highest overall liking score (6.72 ± 1.34), while inulin formulations maintained comparable acceptability up to 50% substitution (6.52–6.88) but decreased thereafter. In contrast, polydextrose formulations showed a sharp decline beyond 50% replacement, with PDX-75 and PDX-100 receiving significantly lower scores (4.80–4.71, *p* < 0.05). This reduction in acceptability can be attributed to the chemical characteristics of these sugar replacers. Their long-chain polymeric structures and numerous hydroxyl groups are known to interact with bitter taste receptors on the tongue [45]. Moreover, partial thermal degradation of these polysaccharides during jelly processing may yield small molecules such as fructose or organic acid derivatives, altering the sweetness–acidity balance compared with sucrose, which provides a clean sweet taste. Consequently, both flavor and textural balance were affected, resulting in distinctive but less-preferred sensory attributes. These findings are consistent with Bolshakova et al. [46], who reported that high levels of polydextrose (≥22.55% of total carbohydrates) increased bitterness in sweetened condensed milk (SCM). Among the tested formulations, chamomile jelly containing 50% inulin exhibited the closest overall liking scores to the control, and was therefore selected for subsequent storage stability evaluation at 4 °C.

#### 3.1.8. Nutrition Profile

The nutritional profile of chamomile jelly formulated with 50% inulin as a sugar substitute (Table 5) was analyzed by the Institute of Food Research and Product Development, Thailand. One serving (90 g) provided 60 kcal, with no energy derived from fat, 3 g of protein, 11 g of total carbohydrates, and essential minerals, including 45 mg sodium and 45 mg potassium. According to the Thai Ministry of Public Health Notification No. 445 (B.E. 2566) on nutrition, this product would qualify as “fat-free,” as its total fat content is less than 0.5 g per serving. In addition, it can also be claimed as “cholesterol-free,” given that cholesterol is below 2 mg and saturated fat does not exceed 2 g per serving. These findings indicated that chamomile jelly with inulin substitution not only meets the criteria for health-oriented foods in terms of low fat and cholesterol-free properties but also provides additional nutritional benefits from inulin, a prebiotic dietary fiber known to promote gut health and support intestinal balance. Such attributes align with current trends in the development of health-conscious food products that integrate taste, quality, and nutritional value.

### 3.2. Phase II: Evaluation of Physicochemical and Functional Changes in Chamomile Jelly During Storage for 21 Days

#### 3.2.1. Chemical, Physical, and Physicochemical Characteristics of Selected Chamomile Jelly Sample

There were only minor changes in the pH values of the jelly samples (Table 6) throughout the storage period. This stability could be attributed to the inherent chemical stability of the major components (chamomile, gelatin, inulin) which are not easily degraded into acidic or alkaline compounds. Furthermore, low storage temperature slowed down chemical reactions such as the Maillard reaction and the degradation of polysaccharides and pectic substances, which may yield acidic intermediates and contribute to slight pH changes, thereby maintaining overall pH stability. Additionally, the low water activity helped inhibit microbial growth and subsequent acid production, which contributed to the overall stability of the jelly quality during storage.

The syneresis values (Table 6) of both the control and the 50% inulin-substituted samples decreased progressively over the storage period at 4 °C, with the highest values observed immediately after production. Initially, the gel network was not in equilibrium, allowing free water to be expelled. However, over time, the gelatin and inulin molecules realigned and formed hydrogen bonds between the hydroxyl groups (-OH), water, and the polymer network, thereby improving the water retention capacity and stabilizing the gel structure. Notably, the inulin-substituted samples (INU-50) had lower syneresis than the control. This effect may be explained by the larger and more complex molecular structure of inulin than sucrose, which provides more hydroxyl groups for hydrogen bonding with water, thus enhancing the gel’s water-holding capacity.

The color parameters (L*, a*, b*), as shown in Table 6 of both the control and inulin-substituted jellies decreased continuously during storage at 4 °C for 21 days. This decline was likely due to the degradation of phenolic compounds and antioxidants in the chamomile, which are sensitive to light, oxygen, and temperature, resulting in fading color. Additionally, non-enzymatic browning reactions such as the Maillard reaction and polyphenol oxidation, although slower at low temperature, could accumulate over time and affect color stability. Structural changes in the gel, such as syneresis and network deterioration, may also alter light scattering and pigment stability, contributing to the significant reduction in brightness and color intensity.

#### 3.2.2. Texture Profile Parameters of Selected Chamomile Jelly Sample

In both the control and INU-50 samples, there were increasing trends in hardness, adhesiveness, springiness, cohesiveness, and gumminess and gel strength during storage (Table 7). This behavior could be attributed to the reinforcement of intermolecular interactions, particularly the formation of hydrogen bonds between the hydroxyl groups (−OH) of sugars or inulin and gelatin chains. In addition, the rearrangement of polymer chains and the stabilization of polymer–water linkages contributed to a denser and more robust gel network. These structural modifications were reflected in the progressive changes in texture-related properties throughout the storage period. These findings were consistent with other reports (Ünal and Arslan [3] and Delgado and Bañón [35] highlighting the role of polysaccharide–protein interactions in modulating gel strength and enhancing texture stability in functional gel systems.

#### 3.2.3. Microbial Loads of Chamomile Jelly of Selected Chamomile Jelly Sample

Microbiological safety in processed foods is critical to consumer health, as inadequate control measures can lead to foodborne illnesses with potentially severe health impacts. The microbial load of a product serves as an indicator of safety and hygiene during production and storage. The presence of pathogenic microorganisms (*Staphylococcus aureus*, *E. coli*, yeasts, and molds) was monitored throughout 21 days of storage at 4 °C, with the results presented in Table 8. The microbiological analysis revealed that *Staphylococcus aureus* was not detected, while the *E. coli* levels remained below 3 MPN/g, and the yeast and mold counts were consistently under 10 CFU/g throughout storage. These values complied with the criteria established for jelly products under the Thai Community Product Standard (TCPS 518/2004: Liquid Jelly), which specifies the maximum permissible limits of ≤1 × 10^4^ CFU/g for total viable loads, ≤100 CFU/g for yeast and molds, and <3 MPN/g for *E. coli*. Additionally, the results were consistent with the Ministry of Public Health Notification No. 416 (2020), which stipulates that no *Salmonella spp*. should be present in 25 g of jelly, and that *Staphylococcus aureus* and *Clostridium perfringens* should not exceed 100 CFU/g [47]. The absence of pathogenic contamination and the low microbial loads observed may be attributed to proper hygienic practices during production, adherence to Good Manufacturing Practice (GMP) guidelines, and the presence of chamomile in the formulation. Chamomile is a rich source of phenolic compounds, which play an important role in inhibiting microbial growth. Consequently, the product consistently met microbiological safety standards throughout the storage period.

### 3.3. Phase III: Evaluation of Biological Properties of Chamomile Jelly Products

#### 3.3.1. In Vitro Prebiotic Activity

**Figure 3:**
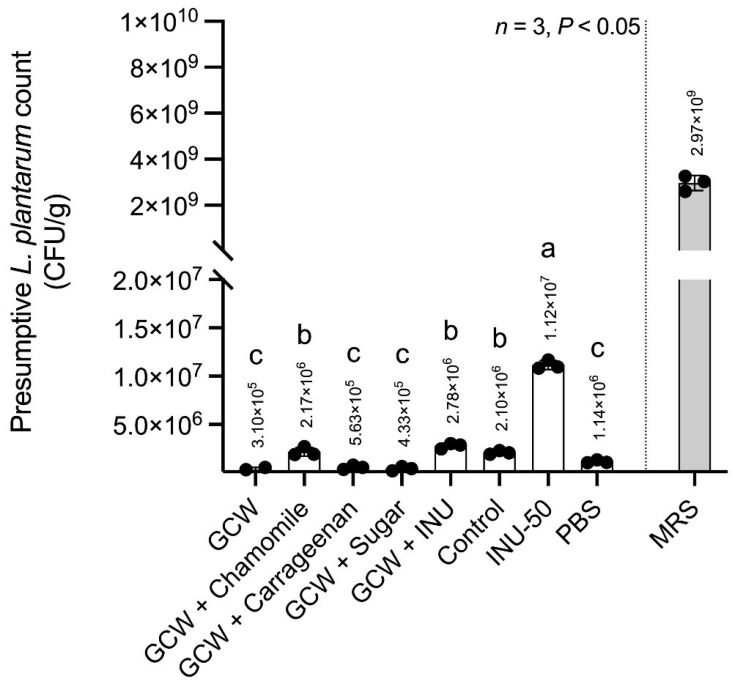
Growth promotion of *Lactobacillus plantarum* TISTR 1465 cultured on media containing different chamomile jelly formulations. Noted: Control (chamomile jellies containing 8.57% sucrose), INU-50 (chamomile jelly containing 4.29% sucrose + 4.29% inulin), GCW (5.15% gelatin capsule waste in water), GCW + Chamomile tea (5.15% GCW in 85.71% chamomile tea), GCW + Carrageenan (5.15% GCW + 0.57% Carrageenan in water), GCW + Sugar (5.15% GCW + 4.29% sucrose in water) and GCW + Inulin (5.15% GCW + 4.29% inulin in water), MRS broth (positive control), and PBS (negative control). MRS refers to De Man, Rogosa, and Sharpe agar, and PBS refers to phosphate-buffered saline. Different lowercase superscripts on the bars indicate statistically significant differences (*p* < 0.05).

The presumptive growth promotion of *Lactobacillus plantarum* grown on media with different samples is presented in Figure 3. The control formulation, containing chamomile tea, gelatin capsule waste, carrageenan, and sugar, supported moderate presumptive *Lactobacillus plantarum* count (~2.10 × 10^6^ CFU/g), reflecting the limited capacity of sucrose as a sole carbon source. GCW + inulin showed a similar presumptive count (~2.78 × 10^6^ CFU/g) to the control. This result can be explained by the fact that inulin acts as a selective carbon source that stimulates moderate presumptive proliferation of *Lactobacillus plantarum*.

In contrast, the INU-50 formulation enhanced the presumptive *Lactobacillus plantarum* count markedly to >1.12 × 10^7^ CFU/g, approximately 5–6 times higher than the control. This effect highlighted the synergistic interaction among the jelly components containing inulin. The combined presence of inulin, chamomile extract, gelatin capsule waste, and carrageenan likely created a favorable microenvironment that supported the metabolic activity of *Lactobacillus plantarum*. In particular, inulin served as a selective carbon source, while other matrix components provided additional nutrients and structural stability, collectively promoting efficient fermentation and biomass accumulation. Other formulations demonstrated that GCW alone or combined with additives (carrageenan and sugar) supported only minimal presumptive growth, while GCW with chamomile tea showed a similar presumptive count to the control, and PBS yielded a negligible presumptive count. MRS broth, as a reference medium, supported the highest presumptive proliferation (10^9^ CFU/g). Based on these results, moderate inulin substitution optimally balances substrate availability and metabolic efficiency, making INU-50 the most effective jelly matrix for probiotic delivery.

#### 3.3.2. Metabolomic Analysis of Postbiotic Compounds Derived from *Lactobacillus plantarum* TISTR 1465 Grown on Chamomile Jelly Substituted with 50% Inulin

**Figure 4:**
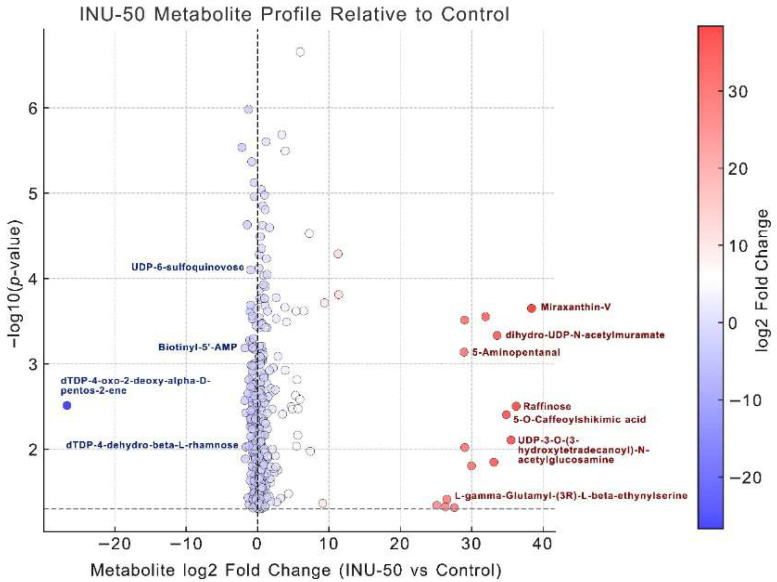
Volcano plot of differential metabolites between Control and INU-50 groups. Each point represents a metabolite detected based on untargeted metabolomic profiling, where *x*-axis indicates log_2_ fold change (Control vs. INU-50) and *y*-axis represents log_10_(*p*-value), circle markers are shaded along blue-white-red gradient, where blue indicates downregulation, red indicates upregulation, and white denotes no major change. Horizontal dashed line corresponds to significance threshold (*p* < 0.05), and vertical dashed line indicates no fold change. Key metabolites highlighted in dark red text (raffinose, UDP-glucose derivatives, 5-aminopentanal, γ-glutamyl compounds, Biotinyl-AMP, dTDP-pentose intermediates, caffeoylshikimic acid) emphasized in discussion due to their roles in carbohydrate metabolism, amino acid biosynthesis, and stress-related pathways. These metabolite shifts mechanistically explain the superior probiotic growth observed in INU-50 formulation.

Metabolomic profiling of the INU-50 group, in which 50% inulin replaced sugar in chamomile jelly, revealed extensive metabolic reprogramming compared with the control, identifying 426 differential metabolites (*p* < 0.05). Among these, the top 20 significantly upregulated and top 20 downregulated metabolites are listed in Table 9 and Table 10, respectively, with the biologically relevant metabolites highlighted in bold. In the upregulated group (Table 9 and Figure 4), metabolites associated with carbohydrate metabolism such as raffinose, L-rhamnono-1,4-lactone, and UDP-N-acetylglucosamine derivatives were markedly elevated, indicating enhanced fermentation of inulin-derived oligosaccharides and activation of the fructose, mannose, and galactose pathways. Amino acid metabolism was also stimulated, as shown by the higher levels of 5-aminopentanal, gabaculine, and L-γ-glutamyl-β-ethynylserine, which contribute to biomass formation and cell proliferation. Secondary metabolites such as Miraxanthin-V, 5-O-caffeoylshikimic acid, and 3-methoxytyramine were also enriched, implying antioxidant-mediated protection and metabolic stability of *L. plantarum* during fermentation. Similar metabolomic shifts have been reported in other antioxidant-enriched or fermented products. For instance, fermentation of apple juice with *Lactobacillus plantarum* ATCC 14,917 led to the upregulation of carbohydrate and secondary metabolism pathways associated with improved antioxidant capacity [48]. Likewise, fermentation of red raspberry juice by *Lacticaseibacillus paracasei* subsp. *paracasei* FBKL1.0328 and *Lactiplantibacillus plantarum* subsp. *plantarum* FBKL1.0310 induced significant alterations in secondary metabolites—including phenolic acids, flavonoids, and alkaloids—accompanied by the activation of flavone/flavonol biosynthesis and tyrosine metabolism pathways, thereby enhancing the overall antioxidant potential [49].

Conversely, Table 10 and Figure 4 list downregulated metabolites, including nucleotide intermediates (e.g., biotinyl-5′-AMP, dTDP-4-oxo-2-deoxy-α-D-pentos-2-ene) and stress-related compounds, suggesting a shift from energy-intensive stress adaptation toward anabolic growth and metabolite biosynthesis. This finding is in line with the report by Fang et al. [50], who observed that in milk fermented with *Lacticaseibacillus paracasei*, the down-regulation of nucleotide intermediates and stress-related compounds may indicate a metabolic shift from energy-intensive stress adaptation toward anabolic growth and biosynthesis. Collectively, these metabolic shifts indicate that inulin substitution in the INU-50 formulation promotes selective prebiotic substrate utilization, stimulates amino acid and secondary metabolite production, reduces oxidative and nutrient stress, and thereby enhances postbiotic compound generation and overall probiotic viability. The enrichment of chamomile-derived antioxidant metabolites further supports this synergistic effect, highlighting INU-50 as a promising prebiotic chamomile tea jelly with functional and probiotic benefits.

## 4. Conclusions

Overall, replacing sucrose with inulin and polydextrose clearly affected the chemical, physical, and functional properties of chamomile jelly. The proximate analysis showed it contained a lower level of carbohydrate but higher fat and ash contents. Samples with inulin and polydextrose had higher water activity, lower pH, darker color, and increased turbidity. Textural properties, such as hardness, cohesiveness, and gel strength, were improved due to the stronger hydrogen bonding and a denser gel network. The antioxidant activity was mainly from chamomile. Consumer acceptance decreased with higher substitution levels, especially with polydextrose, while the 50% inulin formulation achieved the best formulation.

During storage at 4 °C for 21 days, the INU-50 jelly (50% inulin) maintained stable pH, reduced syneresis, and showed gradual changes in color and texture, with microbial counts within safe limits. Metabolomic analysis revealed increased carbohydrate and amino acid metabolism, higher levels of antioxidant-related metabolites, and optimized energy use, supporting a 5–6 fold increase in probiotic growth compared to the control. Nutritional analysis confirmed that the INU-50 jelly was fat-free, cholesterol-free, and enriched with dietary fiber.

In conclusion, the 50% inulin formulation offered the best combination of quality, stability, nutrition, and probiotic benefits, highlighting its potential as a functional prebiotic chamomile jelly suitable for health-conscious consumers. This study highlights the potential of upcycling pharmaceutical gelatin capsule waste into value-added functional food products.

## Figures and Tables

**Table 1:** Formulation of chamomile tea jelly.

Ingredient (%)	Treatment								
	Control	INU- 25	INU- 50	INU- 75	INU- 100	PDX- 25	PDX- 50	PDX- 75	PDX- 100
Chamomile tea	85.71	85.71	85.71	85.71	85.71	85.71	85.71	85.71	85.71
GCW *	5.15	5.15	5.15	5.15	5.15	5.15	5.15	5.15	5.15
Carrageenan	0.57	0.57	0.57	0.57	0.57	0.57	0.57	0.57	0.57
Sugar	8.57	6.43	4.29	2.14	0.00	6.43	4.29	2.14	0.00
Inulin (INU)	-	2.14	4.29	6.43	8.57	-	-	-	-
Polydextrose (PDX)	-	-	-	-	-	2.14	4.29	6.43	8.57

* Note: GCW refers to gelatin capsule waste.

**Table 2:** Proximate composition of chamomile jellies substituted with inulin or polydextrose at different levels.

Samples	Moisture (%)	Fat (%)	Ash (%)	Protein (%)	Carbohydrate (%)
Control	85.14 ± 0.04 ^a^	0.01 ± 0.01 ^c^	0.19 ± 0.01 ^a^	1.67 ± 1.45 ^b^	13.50 ± 1.70 ^a^
INU-25	85.13 ± 0.16 ^a^	0.05 ± 0.03 ^bc^	0.21 ± 0.04 ^a^	2.28 ± 0.03 ^ab^	12.34 ± 0.08 ^bc^
INU-50	85.11 ± 0.04 ^a^	0.08 ± 0.06 ^abc^	0.24 ± 0.06 ^a^	2.10 ± 0.06 ^ab^	12.47 ± 0.15 ^b^
INU-75	85.00 ± 0.36 ^a^	0.04 ± 0.05 ^bc^	0.28 ± 0.09 ^a^	2.28 ± 0.04 ^ab^	12.40 ± 0.34 ^bc^
INU-100	85.17 ± 0.38 ^a^	0.15 ± 0.01 ^a^	0.43 ± 0.35 ^a^	2.45 ± 0.00 ^ab^	11.80 ± 0.57 ^bc^
PDX-25	85.29 ± 0.12 ^a^	0.13 ± 0.03 ^ab^	0.20 ± 0.06 ^a^	2.46 ± 0.03 ^ab^	11.92 ± 0.21 ^bc^
PDX-50	85.15 ± 0.06 ^a^	0.10 ± 0.01 ^abc^	0.35 ± 0.26 ^a^	2.57 ± 0.00 ^ab^	11.82 ± 0.20 ^bc^
PDX-75	85.07 ± 0.05 ^a^	0.10 ± 0.07 ^abc^	0.28 ± 0.11 ^a^	2.49 ± 0.02 ^ab^	12.07 ± 0.05 ^bc^
PDX-100	85.35 ± 0.32 ^a^	0.12 ± 0.09 ^ab^	0.47 ± 0.49 ^a^	2.72 ± 0.02 ^a^	11.35 ± 0.80 ^c^

Note: INU-25, INU-50, INU-75, and INU-100 refer to chamomile jellies in which sugar was substituted with inulin at levels of 25%, 50%, 75%, and 100%, respectively, while PDX-25, PDX-50, PDX-75, and PDX-100 refer to chamomile jellies with sugar substituted by polydextrose at the same levels. Values (mean ± standard deviation). Different lowercase superscripts in same columns indicate statistically significant differences (*p* < 0.05).

**Table 3:** Chemical properties and physicochemical properties of chamomile jellies substituted with inulin or polydextrose at different levels.

Samples	pH	Color			
		L*	a*	b*	ΔE*
Control	7.59 ± 0.17 ^a^	29.01 ± 0.35 ^a^	6.69 ± 0.23 ^cd^	37.29 ± 1.87 ^a^	-
INU-25	7.24 ± 0.02 ^bc^	28.90 ± 0.68 ^a^	6.88 ± 0.42 ^bcd^	35.82 ± 1.49 ^ab^	2.36 ± 1.46 ^b^
INU-50	7.25 ± 0.01 ^bc^	28.74 ± 0.47 ^ab^	6.81 ± 0.37 ^cd^	35.10 ± 2.04 ^b^	2.12 ± 1.42 ^b^
INU-75	7.24 ± 0.03 ^bc^	28.50 ± 0.43 ^abc^	7.50 ± 0.21 ^a^	35.38 ± 2.42 ^b^	2.23 ± 1.15 ^b^
INU-100	7.29 ± 0.01 ^b^	28.16 ± 0.40 ^bc^	6.90 ± 0.22 ^bc^	35.18 ± 1.12 ^b^	2.27 ± 0.72 ^b^
PDX-25	7.18 ± 0.02 ^bcd^	28.00 ± 0.68 ^c^	6.58 ± 0.30 ^de^	31.51 ± 0.70 ^c^	4.59 ± 1.13 ^a^
PDX-50	7.20 ± 0.02 ^bcd^	28.47 ± 1.04 ^abc^	6.64 ± 0.20 ^cde^	31.66 ± 1.50 ^c^	4.87 ± 1.09 ^a^
PDX-75	7.09 ± 0.01 ^d^	28.14 ± 0.94 ^bc^	7.13 ± 0.19 ^b^	32.29 ± 0.96 ^c^	4.82 ± 1.04 ^a^
PDX-100	7.13 ± 0.07 ^cd^	28.77 ± 0.54 ^ab^	6.35 ± 0.41 ^e^	32.77 ± 1.21 ^c^	4.77 ± 0.94 ^a^

Note: INU-25, INU-50, INU-75, and INU-100 refer to chamomile jellies in which sugar was substituted with inulin at levels of 25%, 50%, 75%, and 100%, respectively, while PDX-25, PDX-50, PDX-75, and PDX-100 refer to chamomile jellies with sugar substituted by polydextrose at the same levels. Values (mean ± standard deviation). Different lowercase superscripts in same columns indicate statistically significant differences (*p* < 0.05).

**Table 4:** Texture profile analysis and gel strength of chamomile jellies substituted with inulin or polydextrose at different levels.

Samples	Texture Profile Analysis					Gel Strength/ Bloom Value (g)
	Hardness (N)	Adhesiveness (Nxsec)	Springiness	Cohesiveness	Gumminess (N)	
Control	9.61 ± 0.17 ^c^	−0.83 ± 0.36 ^c^	0.91 ± 0.03 ^bc^	0.76 ± 0.02 ^a^	7.30 ± 0.15 ^c^	54.35 ± 3.24 ^e^
INU-25	9.77 ± 0.35 ^c^	−0.41 ± 0.43 ^ab^	0.94 ± 0.04 ^ab^	0.74 ± 0.02 ^bc^	8.17 ± 1.09 ^bc^	68.15 ± 2.98 ^c^
INU-50	9.79 ± 0.88 ^c^	−0.44 ± 0.20 ^ab^	0.93 ± 0.03 ^bc^	0.71 ± 0.02 ^de^	8.02 ± 1.42 ^bc^	56.59 ± 2.77 ^e^
INU-75	10.08 ± 0.60 ^c^	−0.42 ± 0.27 ^ab^	0.93 ± 0.03 ^bc^	0.72 ± 0.03 ^de^	7.87 ± 0.84 ^bc^	55.97 ± 3.13 ^e^
INU-100	11.08 ± 0.77 ^b^	−0.74 ± 0.38 ^bc^	0.91 ± 0.03 ^c^	0.70 ± 0.04 ^e^	8.03 ± 0.65 ^bc^	63.01 ± 4.38 ^d^
PDX-25	13.46 ± 0.81 ^a^	−0.32 ± 0.40 ^a^	0.97 ± 0.02 ^a^	0.73 ± 0.02 ^bcd^	9.46 ± 0.53 ^a^	81.45 ± 4.56 ^a^
PDX-50	9.83 ± 0.85 ^c^	−0.35 ± 0.17 ^a^	0.94 ± 0.02 ^ab^	0.74 ± 0.02 ^ab^	7.72 ± 1.15 ^bc^	57.34 ± 1.86 ^e^
PDX-75	11.31 ± 0.93 ^b^	−0.48 ± 0.35 ^ab^	0.94 ± 0.03 ^bc^	0.74 ± 0.02 ^bc^	8.37 ± 0.85 ^b^	61.06 ± 2.94 ^d^
PDX-100	10.20 ± 0.46 ^c^	−0.50 ± 0.28 ^ab^	0.93 ± 0.03 ^bc^	0.71 ± 0.02 ^e^	7.61 ± 0.42 ^bc^	72.81 ± 2.37 ^b^

Note: INU-25, INU-50, INU-75, and INU-100 refer to chamomile jellies in which sugar was substituted with inulin at levels of 25%, 50%, 75%, and 100%, respectively, while PDX-25, PDX-50, PDX-75, and PDX-100 refer to chamomile jellies with sugar substituted by polydextrose at the same levels. Values (mean ± standard deviation). Different lowercase superscripts in same columns indicate statistically significant differences (*p* < 0.05).

**Table 5:** Nutritional values of chamomile jelly formulated with 50% inulin as a sugar substitute.

Composition	Content per 100 g	Serving Size (90 g)
Total energy (Kcal)	61.30	60
Total fat (g)	0.06	-
Saturated fatty acids (g)	0.02	-
Cholesterol (mg)	-	-
Protein (g)	2.95	3
Total carbohydrate (g)	12.24	11
Total sugar (g)	6.31	6
Sodium (mg)	48.33	45
Potassium (mg)	56.73	45
Ash (g)	0.20	-
Moisture (g)	84.55	-

The symbol “-” indicates that the value was not detected or is not applicable.

**Table 6:** Chemical and physicochemical properties of chamomile jellies during storage at 4 ± 1 °C for 21 days.

Sample	Day	pH	Syneresis	Color		
				L*	a*	b*
Control	1	7.44 ± 0.02 ^c^	1.70 ± 2.54 ^a^	30.84 ± 2.64 ^bc^	31.68 ± 2.20 ^a^	3.85 ± 0.34 ^a^
	7	7.60 ± 0.05 ^a^	0.57 ± 0.18 ^a^	31.88 ± 3.79 ^ab^	31.26 ± 3.44 ^ab^	3.64 ± 0.53 ^ab^
	14	7.53 ± 0.04 ^ab^	0.13 ± 0.02 ^a^	32.16 ± 1.19 ^ab^	31.01 ± 0.81 ^ab^	3.25 ± 0.80 ^b^
	21	7.50 ± 0.07 ^bc^	0.12 ± 0.26 ^a^	34.45 ± 3.43 ^a^	30.76 ± 1.16 ^ab^	3.25 ± 0.53 ^b^
INU-50	1	7.36 ± 0.05 ^d^	0.28 ± 0.03 ^a^	32.39 ± 1.82 ^ab^	31.68 ± 2.20 ^a^	3.85 ± 0.34 ^a^
	7	7.53 ± 0.01 ^ab^	0.48 ± 0.03 ^a^	29.04 ± 3.65 ^c^	31.26 ± 3.44 ^ab^	3.64 ± 0.53 ^ab^
	14	7.48 ± 0.03 ^bc^	0.22 ± 0.13 ^a^	23.93 ± 1.15 ^e^	31.01 ± 0.81 ^ab^	3.25 ± 0.80 ^b^
	21	7.46 ± 0.01 ^bc^	0.21 ± 0.10 ^a^	26.53 ± 2.41 ^d^	30.76 ± 1.16 ^ab^	3.25 ± 0.53 ^b^

Note: Different lowercase superscripts in same column indicate significant difference (*p* < 0.05). INU-50 refers to chamomile jellies in which sugar was replaced with 50% inulin.

**Table 7:** Texture profile analysis and gel strength of chamomile jellies during storage at 4 ± 1 °C for 21 days.

Samples	Day	Texture Profile Analysis					Gel Strength/ Bloom Value (g)
		Hardness (N)	Adhesiveness (Nxsec)	Springiness	Cohesiveness	Gumminess (N)	
Control	1	3.90 ± 0.70 ^d^	−0.56 ± 0.23 ^bc^	0.81 ± 0.05 ^b^	0.27 ± 0.04 ^e^	1.04 ± 0.13 ^f^	64.80 ± 7.04 ^d^
	7	4.37 ± 0.43 ^d^	−0.62 ± 0.30 ^d^	0.85 ± 0.03 ^b^	0.26 ± 0.02 ^e^	1.15 ± 0.13 ^f^	65.80 ± 7.58 ^d^
	14	8.80 ± 0.74 ^b^	−0.27 ± 0.22 ^b^	0.94 ± 0.03 ^ab^	0.56 ± 0.09 ^b^	4.99 ± 1.09 ^c^	69.79 ± 6.43 ^bcd^
	21	9.47 ± 0.89 ^b^	−0.33 ± 0.37 ^bc^	1.09 ± 0.47 ^a^	0.70 ± 0.04 ^a^	6.66 ± 0.93 ^b^	88.89 ± 6.42 ^a^
INU-50	1	4.32 ± 0.50 ^d^	−0.48 ± 0.37 ^bc^	0.87 ± 0.05 ^b^	0.37 ± 0.05 ^d^	1.58 ± 0.31 ^f^	67.16 ± 4.58 ^cd^
	7	6.93 ± 0.84 ^c^	0.07 ± 0.01 ^a^	0.89 ± 0.04 ^b^	0.34 ± 0.07 ^d^	2.38 ± 0.60 ^e^	71.87 ± 4.00 ^bc^
	14	8.99 ± 1.11 ^b^	−0.46 ± 0.30 ^bc^	0.94 ± 0.02 ^ab^	0.45 ± 0.15 ^c^	4.15 ± 1.70 ^d^	73.24 ± 3.87 ^b^
	21	10.25 ± 0.70 ^a^	−0.49 ± 0.36 ^bc^	0.95 ± 0.05 ^ab^	0.75 ± 0.02 ^a^	7.73 ± 0.54 ^a^	88.78 ± 6.39 ^a^

Note: Different lowercase superscripts in same column indicate significant difference (*p* < 0.05). INU-50 refers to chamomile jellies in which sugar was replaced with 50% inulin.

**Table 8:** Microbiological load of chamomile jellies during storage at 4 ± 1 °C for 21 days.

Sample	Day	Parameter			
		*Staphylococcus aureus* (CFU/g)	*E. coli* (MPN/g)	*Yeasts* (CFU/g)	*Molds* (CFU/g)
Control	1	ND	<3	<10	<10
	7	ND	<3	<10	<10
	14	ND	<3	<10	<10
	21	ND	<3	<10	<10
INU-50	1	ND	<3	<10	<10
	7	ND	<3	<10	<10
	14	ND	<3	<10	<10
	21	ND	<3	<10	<10

Note: There were no significant differences (*p* < 0.05). ND: Not detected. INU-50 refers to chamomile jellies in which sugar was replaced with 50% inulin.

**Table 9:** Upregulated metabolites between control and INU-50.

No.	Name	Fold-Change (FC)	log_2_FC	*p*-Value
Up Regulation				
**1**	Miraxanthin-V	358,675,324,476.240	38.3839	0.0002
**2**	Raffinose **	80,710,592,233.243	36.2320	0.0031
**3**	dihydro-UDP-N-acetylmuramate **	48,596,078,146.530	35.5001	0.0078
**4**	5-O-Caffeoylshikimic acid **	30,955,125,405.091	34.8495	0.0039
**5**	(S)-3-Hydroxyhexadecanoyl-CoA	12,674,769,966.638	33.5612	0.0005
**6**	UDP-3-O-(3-hydroxytetradecanoyl)-N-acetylglucosamine **	9,241,193,149.530	33.1054	0.0142
**7**	L-Rhamnono-1,4-lactone	4,126,665,535.711	31.9423	0.0003
**8**	Cobalt-precorrin 7	1,058,583,229.174	29.9795	0.0157
**9**	gabaculine	545,654,526.282	29.0234	0.0096
**10**	5-Aminopentanal **	537,965,022.664	29.0029	0.0003
**11**	3-Methoxytyramine	508,790,935.368	28.9225	0.0007
**12**	L-gamma-Glutamyl-(3R)-L-beta-ethynylserine **	198,930,782.304	27.5677	0.0485
**13**	2-Amino-5-oxohexanoate	96,834,576.078	26.5290	0.0386
**14**	2-Phenyl-1,3-propanediol monocarbamate	83,061,174.704	26.3077	0.0474
**15**	Aminoadipic acid	36,331,460.343	25.1147	0.0455
**16**	Maltotetraose	2621.107	11.3560	0.0002
**17**	Maltohexaose	2500.159	11.2878	0.0001
**18**	N2-Citryl-N6-acetyl-N6-hydroxy-L-lysine	661.468	9.3695	0.0002
**19**	Isonocardicin A	559.952	9.1292	0.0432
**20**	N-amino DAP	168.248	7.3944	0.0106

Note: ** refers to metabolites highlighted in bold are those discussed in the Results and Discussion section due to their biological relevance.

**Table 10:** Downregulated metabolites between control and INU-50.

No.	Name	Fold-Change	log_2_FC	*p*-Value
Down Regulation				
**1**	dTDP-4-oxo-2-deoxy-alpha-D-pentos-2-ene **	0.000	−26.6906	0.0031
**2**	1-OH-Nogalamycinone	0.220	−2.1837	0.0000
**3**	dTDP-4-dehydro-beta-L-rhamnose **	0.293	−1.7728	0.0027
**4**	(+)-Gallocatechin	0.293	−1.7708	0.0007
**5**	7,8-Dihydromethanopterin	0.303	−1.7215	0.0130
**6**	Arachidonyl-CoA	0.315	−1.6650	0.0037
**7**	Baicalin	0.321	−1.6388	0.0094
**8**	Tetrahydrosarcinapterin	0.370	−1.4353	0.0000
**9**	UDP-6-sulfoquinovose **	0.395	−1.3417	0.0372
**10**	Sucrose	0.396	−1.3365	0.0022
**11**	Ethyl-D-glucuronide	0.424	−1.2392	0.0000
**12**	Hygromycin B	0.430	−1.2189	0.0092
**13**	N1-Amidinostreptamine 6-phosphate	0.435	−1.2001	0.0015
**14**	CDP-DG (16:0/20:4(8Z,11Z,14Z,17Z))	0.447	−1.1625	0.0446
**15**	Biotinyl-5′-AMP **	0.466	−1.1019	0.0005
**16**	Acarbose 7IV-phosphate	0.480	−1.0598	0.0234
**17**	Glycolate	0.481	−1.0548	0.0002
**18**	Ampicillin	0.490	−1.0282	0.0054
**19**	N-Methylethanolamine phosphate	0.505	−0.9856	0.0045
**20**	4-Amino-4-deoxychorismate	0.511	−0.9695	0.0001

Note: ** refers to metabolites highlighted in bold are those discussed in the Results and Discussion section due to their biological relevance.

## Data Availability

Data are contained within the article and Supplementary Materials.

## Supplementary Materials

The following supporting information can be downloaded at: https://www.mdpi.com/article/10.3390/antiox14111380/s1, Table S1: Water activity (aw) and antioxidant activity of chamomile jellies substituted with inulin or polydextrose at different levels.
