# Supplementary material for: Functional and Metabolomic Analyses of Chamomile Jelly Derived from Gelatin Capsule Waste with Inulin and Polydextrose as Prebiotic Sugar Substitutes
Source: Antioxidants (Basel). 2025 Nov 19;14(11):1380. doi: 10.3390/antiox14111380 (PMC12649176; doi:10.3390/antiox14111380)
Supplement: Supplementary file 1 [file antioxidants-14-01380-s001.zip › antioxidants-3949799-supplementary.pdf]

antioxidants

MDPI

Article

Functional and Metabolomic Analyses of Chamomile Jelly Derived from Gelatin Capsule Waste with Inulin and Polydextrose as Prebiotic Sugar Substitutes

Sasina Sanprasert [1], Anurak Uchuwittayakul [2], Pudthaya Kumnerdsiri [1], Lalitphan Kitsanayanyong [1], Anusorn Seubsai [3], Jaksuma Pongsetkul [4], Kantiya Petsong [5], Supatra Karnjanapratum [6], Chalalai Jaisan [7], Samart Sai-ut [8], Saroat Rawdkuen [9] and Passakorn Kingwascharapong [1,*]

[1] Department of Fishery Products, Faculty of Fisheries, Kasetsart University, Bangkok 10900, Thailand; sasina.sanp@ku.th (S.S.); pudthaya.k@ku.th (P.K.); ffislhk@ku.ac.th (L.K.)
[2] Department of Aquaculture, Faculty of Fisheries, Kasetsart University, Bangkok 10900, Thailand; ffisarb@ku.ac.th
[3] Department of Chemical Engineering, Faculty of Engineering, Kasetsart University, Bangkok 10900, Thailand; fengasn@ku.ac.th
[4] School of Animal Technology and Innovation, Institute of Agricultural Technology, Suranaree University of Technology, Nakhon Ratchasima 30000, Thailand; jaksuma@sut.ac.th
[5] Department of Food Technology, Faculty of Technology, Khon Kaen University, Khon Kaen, 40002, Thailand; kantpe@kku.ac.th
[6] Faculty of Agro-Industry, Chiang Mai University, Chiang Mai, 50100, Thailand; supatra.ka@cmu.ac.th (S.K.)
[7] Faculty of Agro-Industry, Chiang Mai University, Samut Sakhon 74000, Thailand; chalalai.jai@cmu.ac.th (C.J.)
[8] Department of Food Science, Faculty of Science, Burapha University, Chonburi 20131, Thailand; samarts@go.buu.ac.th
[9] Unit of Innovative Food Packaging and Biomaterials, School of Agro-Industry, Mae Fah Luang University, Chiang Rai 57100, Thailand; saroat@mfu.ac.th
* Correspondence: passakorn.ki@ku.th

 https://doi.org/10.3390/antiox14111380

Supplementary Table S1. Water activity (aw) and antioxidant activity of chamomile jellies substituted with inulin or polydextrose at different levels.

Sample	a_w	Total phenolic content (mg GAE/g sample)	ABTS radical scavenging activity (μmol TE/g sample)	DPPH radical scavenging activity (μmol TE/g sample)
Control	0.9822±0.0007[b]	137.88±1.61[a]	2.75±0.36[a]	0.12±0.02[a]
INU-25	0.9863±0.0003[a]	135.30±1.61[a]	2.69±0.04[a]	0.13±0.01[a]
INU-50	0.9859±0.0004[a]	133.96±2.95[a]	2.65±0.07[a]	0.12±0.01[a]
INU-75	0.9850±0.0005[a]	133.69±5.51[a]	2.64±0.10[a]	0.13±0.02a
INU-100	0.9856±0.0004[a]	135.14±2.66[a]	2.65±0.04[a]	0.11±0.01[a]
PDX-25	0.9868±0.0001[a]	135.94±3.80[a]	2.74±0.04[a]	0.13±0.01[a]
PDX -50	0.9867±0.0008[a]	135.30±1.29[a]	2.72±0.04[a]	0.12±0.00[a]
PDX -75	0.9855±0.0017[a]	135.62±0.97[a]	2.85±0.09[a]	0.13±0.00[a]
PDX -100	0.9863±0.0002[a]	133.80±1.62[a]	2.63±0.08[a]	0.11±0.01[a]

Note: INU-25, INU-50, INU-75, and INU-100 refer to chamomile jellies in which sugar was substituted with inulin at levels of 25%, 50%, 75%, and 100%, respectively, while PDX-25, PDX-50, PDX-75, and PDX-100 refer to chamomile jellies with sugar substituted by polydextrose at the same levels. Values (mean ± standard deviation). Different lowercase superscripts in same columns indicate statistically significant differences ($p < 0.05$).
